# Exploratory Prospective Randomized Controlled Study to Evaluate a Modified Botulinum Toxin Injection Pattern for Treatment of Benign Essential Blepharospasm

**DOI:** 10.7759/cureus.76905

**Published:** 2025-01-04

**Authors:** Robert J Contento, Collin J Anderson, Robert H Hill, Bryant P Carruth, George Salloum, Thomas A Bersani

**Affiliations:** 1 Norton College of Medicine, Upstate University Hospital, Syracuse, USA; 2 Ophthalmology, Upstate University Hospital, Syracuse, USA

**Keywords:** benign essential blepharospasm, botulinum toxin, diplopia, dry eye, eyelid closure, lagophthalmos, orbicularis oculi, pretarsal injection pattern, ptosis, spasm control

## Abstract

Purpose: This study aimed to explore trends in duration of effect, effectiveness of spasm control, and complication rates, as well as assess feasibility between an established versus a modified pretarsal botulinum toxin (Botox) injection pattern for treatment of benign essential blepharospasm (BEB).

Methods: In this exploratory randomized controlled trial, patients diagnosed with BEB (excluding those with prior myectomy) were randomized to receive either an established or modified pretarsal Botox injection pattern on each side of the face. A total of 50 or 60 units of onabotulinumtoxinA (Allergan, Irvine, CA, USA) were administered. Primary outcomes included the duration of effect, spasm control, and complication rates, assessed using a 10-point Likert scale immediately following initial injection and at a three-month follow-up post injection. Statistical analysis was performed using paired t-tests (α<0.05).

Results: Eight female patients (mean age 71.0±9.53 years) were followed for 110±26.9 days. The trends of the results suggest that the modified pattern has a longer mean duration of effect (82.9±38.4 vs. 63.8±36.2 days, p=0.13) and improved spasm control (7.38±2.13 vs. 6.63±2.13, p=0.41). Spasm severity and pain levels during injection were similar between patterns. Complications were comparable, with reports of dry eye, tearing, and ptosis in both groups. Statistical significance was not reached due to small sample size as this was an exploratory study to inform future research.

Conclusion: The modified pretarsal botulinum toxin injection pattern demonstrates potential as an alternative approach for treating BEB, with observed trends toward improved duration and effectiveness of spasm control, without added complications. Further research with larger sample sizes is needed to confirm these preliminary findings.

## Introduction

Benign essential blepharospasm (BEB) is a focal dystonic disease characterized by involuntary contractions of the orbicularis oculi muscles, leading to an increased frequency of bilateral eyelid closure [1,2]. Botulinum toxin injection has been widely recognized as an effective treatment for BEB since its first use in 1984 by Freuh et al. [2]. The location of injection sites has been adapted after its initial adoption via the use of electromyography to determine optimal injection sites [3]. The botulinum toxin inhibits the release of acetylcholine at the neuromuscular junction, preventing contraction of the orbicularis oculi muscle and alleviating eyelid spasms. The efficacy of botulinum toxin injection typically lasts three to four months, although some patients may experience longer periods of relief, while others may require more frequent injections [4]. However, there are side effects associated with botulinum toxin injection, such as diplopia (double vision), blurred vision, increased lacrimation, lagophthalmos, and ptosis [5]. Treatment of BEB includes a variety of injection sites, including the lateral canthi, lateral upper and lower eyelid margins, and the medial upper eyelid margin [6]. Despite the effectiveness of botulinum toxin injection, there has been a wide variation in the duration and degree of spasm control and in the complications reported [7]. The purpose of this study is to explore trends in duration of effect, effectiveness of spasm control, and complication rates, as well as assess feasibility between an established versus a modified pretarsal botulinum toxin (Botox) injection pattern for treatment of BEB. The observed trends of this study will help guide future research to determine the optimal injection pattern for the treatment of BEB, potentially improving patient outcomes and minimizing complications.

## Materials and methods

**Figure 1:**
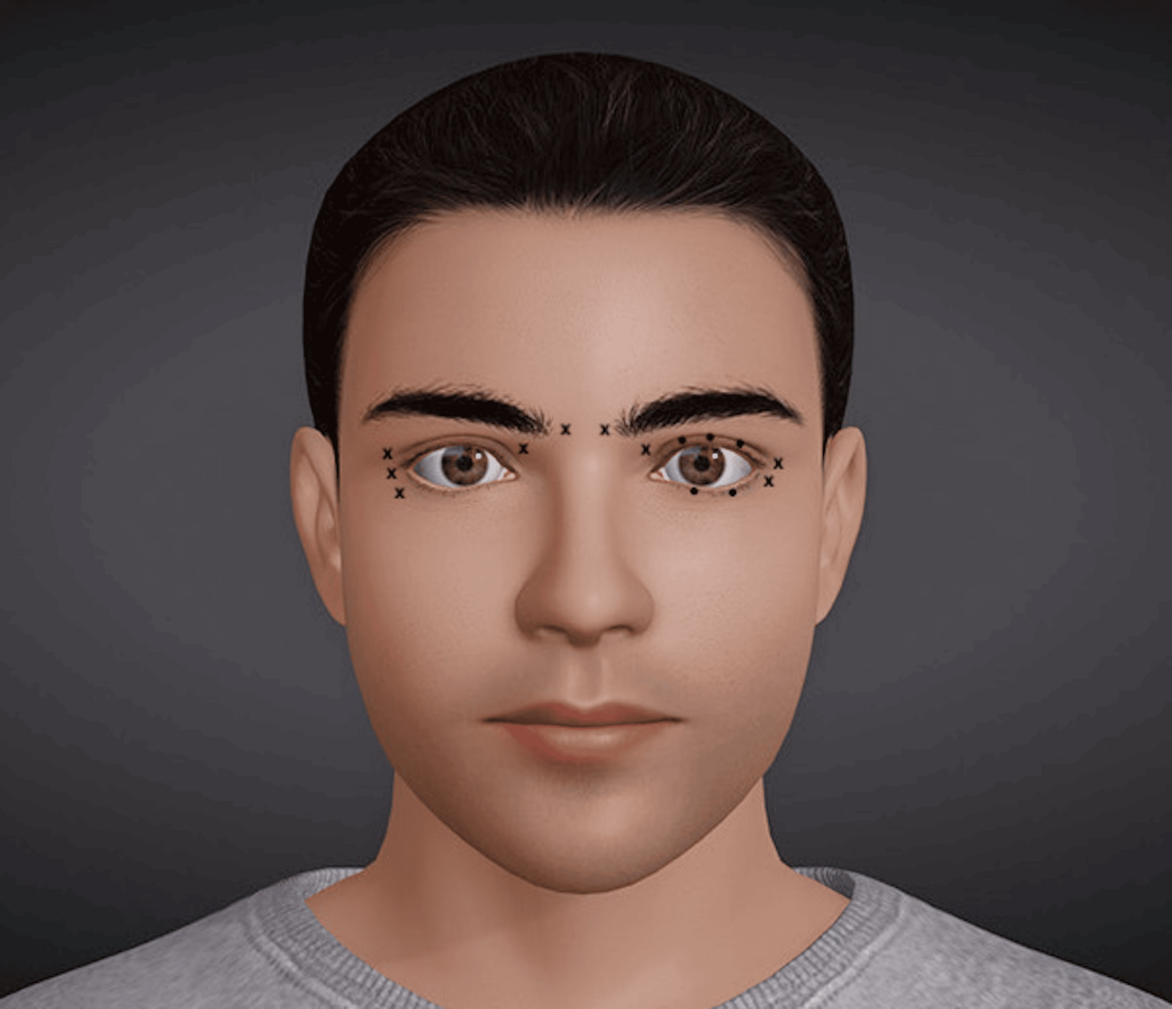
Established pattern (right eye) versus modified pattern (left eye), where pretarsal orbicularis around the eyelid margin is treated. (x) = 5 units (0.20 mL), (•) = 1 unit (0.04 mL) of Botox injection. Credit: Adapted figure from Wikimedia Commons https://commons.wikimedia.org/wiki/File:3D_head_position_model.jpg

In this exploratory randomized controlled study, we evaluated the efficacy of two Botox injection patterns for treating BEB. The first pattern had been a standard in our practice for 35 years, using onabotulinumtoxinA (Allergan, Irvine, CA, USA). The established pattern consisted of injections in the lateral canthal region, medial upper orbicularis oculi, and the medial glabella. The second pattern was a modified approach, where slightly less toxin was injected into the lateral canthal region, and instead, more was directed into the pretarsal upper and lower lids [5,6]. The total Botox dosage administered was approximately 50 units (U), with 25 U allocated to each side of the face. Botox was reconstituted by adding 4 mL of saline to a 100-unit vial, resulting in a final concentration of 2.5 units per 0.1 mL. Of note, two patients received a slightly higher dose of 60 U total (30 U per side), though the relative proportion of the Botox delivered to the eyelid margin was consistent across all participants (Figure 1).

The study enrolled patients who were clinically diagnosed with BEB, excluding those who had previously undergone myectomy. As this was an exploratory study, a formal power calculation was not performed. However, the sample size was deemed appropriate to identify preliminary patterns and inform future research. Each participant was randomly assigned to receive the established injection pattern on one side of their face and the modified pattern on the other side. Randomization was performed to reduce intra-individual variability, allowing each participant to serve as their own control.

The primary outcomes assessed were the duration of effect, the effectiveness of spasm control, and the incidence of complications. These outcomes were measured using a follow-up questionnaire rated on a Likert 10-point scale at the patient’s three-month follow-up appointment. All patient responses were recorded within a three-month interval. Statistical analysis was performed using two-tailed paired samples t-tests to compare mean differences between the two injection patterns (a<0.05).

This study was conducted in accordance with the Health Insurance Portability and Accountability Act (HIPAA) regulations to ensure the confidentiality and protection of patient information. All research procedures adhered to the principles outlined in the Declaration of Helsinki, ensuring ethical treatment and protection of human subjects. The study protocol was reviewed and approved by the Institutional Review Board (IRB) at State University of New York (SUNY) Upstate Medical University, Syracuse, NY, with informed consent obtained from all participants prior to their inclusion in the study (Project # 2015917-5).

## Results

A total of eight female patients were enrolled in the study, with a mean age of 71.0±9.53 years. The mean follow-up period was 110±26.9 days (Table 1). The results showed that the mean duration of effect for the botulinum toxin injections was longer for the modified pretarsal pattern (82.9±38.4 days) compared to the established pattern (63.8±36.2 days), though this difference was not statistically significant (p=0.13). When evaluating the effectiveness of spasm control on a 10-point scale, the modified pattern scored slightly higher (7.38±2.13) than the established pattern (6.63±2.13), but this difference also did not reach statistical significance (p=0.41).

**Table 1:** Patient Characteristics F = female, N = number of patients, SD = standard deviation

	N=8, %
Sex (F)	8 (100)
	(Mean ± SD)
Age (Years)	71.0 ± 9.53
Time to follow-up (days)	110 ± 26.9

In terms of spasm severity immediately after injection, the mean scores were lower for the modified pattern (3.13±2.47) compared to the established pattern (4.13±3.04), and this trend persisted at follow-up, with severity scores of 4.75±3.37 for the modified pattern and 6.75±3.20 for the established pattern. However, these differences were not statistically significant (p=0.17 immediately after injection, p=0.12 at follow-up). Pain levels during the injection were reported to be slightly higher for the modified pattern (4.63±3.25) compared to the established pattern (2.63±2.20), which approached statistical significance (p=0.09) (Table 2). A preference for the modified pattern was also noted, with half of the eight participants opting to adopt the modified approach permanently.

**Table 2:** Comparison Between Established and Modified Pattern

	Established	Modified	
	(Mean ± SD)	(Mean ± SD)	p-value
Duration of effect (days)	63.8 ± 36.2	82.9 ± 38.4	0.13
Effectiveness (0-10)	6.63 ± 2.13	7.38 ± 2.13	0.41
Spasm severity immediately (0-10)	4.13 ± 3.04	3.13 ± 2.47	0.17
Spasm severity at follow-up (0-10)	6.75 ± 3.20	4.75 ± 3.37	0.12
Pain at time of injection (0-10)	2.63 ± 2.20	4.63 ± 3.25	0.09

Complications were comparable between the two injection patterns. In the established pattern, five patients reported dry eye, four experienced tearing, and two developed ptosis. The ptosis observed in these cases, as reported through patient questionnaires, was mild, transient, and clinically insignificant. Importantly, it did not impair visual function or necessitate modifications to the injection protocol. In the modified pattern, six patients reported dry eye, three experienced tearing, and one developed ptosis (Table 3).

**Table 3:** Complications Between Established and Modified Pattern

	Established	Modified
	(N=8)	(N=8)
Dry Eye	5	6
Tearing	4	3
Diplopia	0	0
Ptosis	2	1

## Discussion

This exploratory study suggests potential benefits of a modified pretarsal botulinum toxin injection pattern in managing BEB. Although the results did not reach statistical significance, likely due to the study’s small sample size, the observed trends indicate that the modified pattern may provide a longer duration of effect and slightly better spasm control without significantly increasing the risk of complications compared to the established pattern. Notably, the permanent adoption of the modified pattern by half of our participants further supports these trends. Our findings are consistent with prior studies emphasizing the importance of injection site selection in optimizing treatment outcomes for BEB.

Lolekha et al. demonstrated that low-dose pretarsal injections provide superior efficacy and higher patient satisfaction with fewer complications compared to preseptal injections [8]. This finding reinforces the growing consensus that pretarsal injections may be an optimal strategy for reducing side effects while maintaining treatment effectiveness. Similarly, the American Academy of Ophthalmology emphasized the advantages of pretarsal injections, noting their higher response rates and longer symptom relief durations compared to preseptal patterns [9]. These findings strongly support the rationale for focusing on pretarsal modifications, as employed in our study.

Hu et al. recently published a prospective study that demonstrated a combined pretarsal (PT) and preseptal (PS) botulinum toxin injection approach significantly improved outcomes in BEB patients [10]. Their method reduced onset time, extended the duration of efficacy, and increased patient satisfaction compared to PT injections alone. These results are closely aligned with our study’s outcomes, where a modified pretarsal injection pattern demonstrated a longer duration of effect and improved spasm control while maintaining safety.

Price et al. previously reported that their standard injection pattern, which avoided pretarsal sites, achieved a longer duration of effect compared to alternative patterns such as brow, inner orbital, and outer orbital injections [6]. However, this approach was associated with increased ocular irritation and epiphora. By contrast, our study’s modified injection pattern included upper lid pretarsal injections and achieved trends suggesting improved spasm control without increasing complications such as ptosis. The safety profile of the modified pretarsal injection pattern in our study was comparable to the traditional approach, with trends suggesting no significant increase in complications such as ptosis, diplopia, or dry eye. This is consistent with a recent prospective study done by Hu et al., who found that PT-PS injections offered enhanced outcomes without additional safety concerns, but contrasts the original study done by Price et al. [6,10]. The absence of significant complications in both our study and Hu et al. strengthens the case for integrating pretarsal modifications into clinical practice as a viable alternative to traditional approaches. However, due to the exploratory nature of our study and lack of statistical significance, future research is needed to confirm these trends.

## Conclusions

The modified botulinum toxin injection pattern presents a potential alternative to the established approach for treating benign essential blepharospasm. While this small sample size did not yield statistically significant differences in the duration of effect and spasm control, the results suggest trends toward longer-lasting relief and improved patient satisfaction without increasing complication rates. Notably, half of the eight participants opted to adopt the modified approach permanently, highlighting the clinical significance of even modest improvements in treatment efficacy.

Future research should explore these trends and focus on larger, more diverse cohorts and incorporate objective measures, such as electromyography, to provide a comprehensive assessment of spasm control and muscle activity. Confirming these preliminary results in larger studies could help ophthalmologists integrate the modified injection pattern into practice, potentially offering patients a pathway to longer-lasting symptom relief and better patient care. Importantly, this preliminary data on our modified injection pattern is still considered experimental and should not be adopted until further research confirms our trends.
